# Clinical and Pathophysiological Interplay Between Patent Ductus Arteriosus and Patent Foramen Ovale: A Comparative Narrative Review

**DOI:** 10.1002/vms3.71179

**Published:** 2026-08-18

**Authors:** Amir Mohammad Barati

**Affiliations:** ^1^ Department of Veterinary Medicine Shahid Bahonar University of Kerman Kerman Iran

**Keywords:** congenital heart disease, patent ductus arteriosus, patent foramen ovale, pulmonary hypertension, veterinary cardiology

## Abstract

**Background:**

Patent ductus arteriosus (PDA) and patent foramen ovale (PFO) are two fetal cardiovascular shunts that normally undergo functional and anatomical closure shortly after birth. While PDA is a well‐recognized congenital defect in veterinary cardiology, the haemodynamic interaction between PDA and PFO has received little attention, particularly in neonates.

**Objectives:**

To review the physiological and pathophysiological relationship between PDA and PFO and evaluate available evidence from veterinary and comparative human literature.

**Review Content:**

Increased pulmonary blood flow and secondary pulmonary hypertension associated with PDA may alter right‐sided cardiac pressures and predispose affected neonates to persistent or reopened PFO, potentially resulting in interatrial shunting and cyanosis. PDA represents one of the most prevalent congenital cardiac defects encountered in canine medicine and constitutes a substantial proportion of congenital heart diseases in dogs. Conversely, PFO appears to be less frequently recognized in veterinary patients, likely because many affected animals remain clinically asymptomatic. In human medicine, persistence of the foramen ovale has been identified in approximately one‐quarter of the adult population. This narrative review summarizes the physiology and pathophysiology of ductus arteriosus and foramen ovale closure, analyses the mechanisms by which PDA can influence FO patency, and discusses relevant evidence from both veterinary and human literature.

**Conclusions:**

Although no clearly documented veterinary case of concurrent PDA and PFO has been identified, comparative evidence suggests that secondary pulmonary hypertension may play an important role in this interaction. Improved understanding of this relationship may enhance diagnostic accuracy and management strategies in neonatal animals.

## Graphical abstract

Patent ductus arteriosus may contribute to persistent or reopened patent foramen ovale through secondary pulmonary hypertension and altered right‐sided cardiac pressures. Although concurrent PDA and PFO have not been clearly documented in veterinary medicine, comparative evidence from human literature supports a potential haemodynamic interaction between these congenital cardiovascular shunts. Improved recognition of this relationship may enhance diagnostic evaluation and clinical management in neonatal patients.

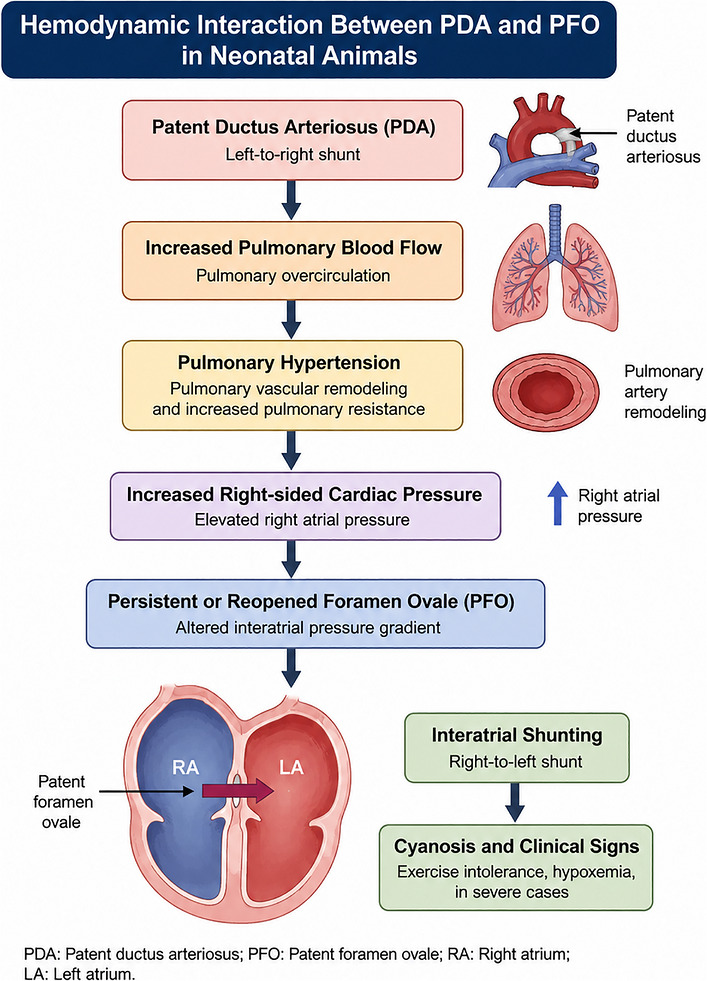

## 1 Introduction

Fetal circulation relies on two primary shunts, the ductus arteriosus (DA) and foramen ovale (FO), which bypass pulmonary flow and allow efficient oxygen delivery from the placenta. Following birth, dramatic haemodynamic changes initiate their closure. Although PDA is a common congenital defect in small animals and livestock, persistent foramen ovale (PFO) is less frequently diagnosed but may contribute to clinically relevant intracardiac shunting.

The interaction between PDA and PFO has been well described in human neonatal cardiology; however, data in veterinary species remain limited. This narrative review outlines the physiological basis of DA and FO closure and evaluates how PDA can influence FO patency in neonatal animals.

The coexistence of PDA and PFO has been described infrequently in human medicine and is generally associated with altered haemodynamic conditions, particularly pulmonary hypertension or elevated right‐sided cardiac pressures.

## 2 Physiology of Ductus Arteriosus and Foramen Ovale Closure

### 2.1 Ductus Arteriosus

During fetal life, the DA diverts blood from the pulmonary artery to the aorta. After birth:

1. **Rise in arterial oxygen tension (PaO_2_)** stimulates constriction of ductal smooth muscle—*the primary mechanism* of functional closure.
2. **Decreased circulating prostaglandin E2 (PGE_2_)** level due to the loss of placental supply promotes further constriction.
3. **Bradykinin**, released from the expanding lungs, may contribute to early ductal contraction, although its effect is modest and species‐dependent.
4. Anatomical closure typically occurs within days to weeks via intimal proliferation.

Failure of these processes results in a PDA with left‐to‐right shunting.

### 2.2 Foramen Ovale

FO allows right‐to‐left shunting of oxygenated umbilical blood into the left atrium during fetal development. After birth:

1. **Pulmonary expansion** reduces pulmonary vascular resistance (PVR).
2. **Increased pulmonary venous return** raises left atrial (LA) pressure.
3. **Right atrial (RA) pressure falls**, causing the septum primum to seal the FO flap.

Closure is initially *functional* but may remain *anatomically incomplete*, creating a PFO.

In humans, 20%–25% of adults retain PFO; in veterinary species, similar persistence is suspected though underreported.

## 3 Pathophysiological Interaction Between PDA and PFO

The haemodynamic consequences of PDA are primarily characterized by a left‐to‐right shunt, increased pulmonary blood flow, and volume overload affecting the left atrium and left ventricle. In the early‐stage of PDA, RA pressure generally remains within normal limits.

However, chronic pulmonary overcirculation may lead to pulmonary vascular remodelling and secondary pulmonary hypertension. Increased RA pressure usually develops in advanced cases or in situations involving shunt reversal (Eisenmenger‐like physiology). Under these conditions, altered interatrial pressure gradients may contribute to persistence or reopening of the FO.

Therefore, the interaction between PDA and PFO is more appropriately attributed to pulmonary hypertension secondary to PDA rather than to PDA alone.

## 4 Clinical and Diagnostic Findings

Patients with PDA commonly present with a continuous ‘machinery’ cardiac murmur, bounding femoral pulses, tachycardia, exercise intolerance and signs of left‐sided volume overload. In advanced or untreated cases, pulmonary hypertension and congestive heart failure may develop. In cases of reversed PDA (Eisenmenger‐like physiology), differential cyanosis and exercise intolerance may also be observed.

Electrocardiographic findings in PDA may include left ventricular enlargement, LA enlargement, and increased QRS amplitude associated with volume overload. Thoracic radiography commonly demonstrates cardiomegaly, enlargement of the pulmonary vasculature and pulmonary overcirculation.

In contrast, PFO is often clinically silent unless associated with significant interatrial shunting or concurrent cardiovascular abnormalities. In some cases, intermittent hypoxemia or cyanosis may occur secondary to altered intracardiac pressure gradients.

Echocardiography remains the gold standard diagnostic modality for both PDA and PFO. PDA is typically identified by continuous turbulent ductal flow, enlargement of the left atrium and left ventricle and abnormal pulmonary arterial flow patterns. In contrast, identification of PFO may require Doppler echocardiography or contrast‐enhanced echocardiographic studies to demonstrate interatrial shunting.

## 5 Evidence From Veterinary Literature

Despite PDA being one of the most common congenital cardiac anomalies in dogs and livestock, no clearly documented veterinary case of concurrent PDA and PFO in the same patient has been identified.

The following findings support this conclusion:

1. Mampaey et al. (2023) described a Doberman puppy with PDA and pulmonary arteriovenous malformation (PAVM); no PFO was detected.
2. Fujii et al. (2009) reported vascular anomalies mimicking PDA, but without FO involvement.
3. Adams et al. (1951) report in the *British Veterinary Journal* documented PFO in a calf, but without coexisting PDA.

A review of textbooks, conference proceedings, and peer‐reviewed literature (PubMed, CAB Abstracts and Google Scholar search through July 2025) revealed *no confirmed cases* of simultaneous PDA and PFO.

Human studies suggest that pulmonary hypertension and elevated right‐sided cardiac pressures may facilitate interatrial shunting through a patent FO. These observations provide a valuable comparative framework for understanding similar pathophysiological mechanisms in veterinary patients with advanced PDA.

This absence may reflect rarity, underdiagnosis or limited use of advanced imaging in neonates.

## 6 Discussion

Theoretical and human‐based evidence strongly supports the possibility that haemodynamic consequences of PDA can promote FO persistence. Veterinary neonates—especially those with severe PDA causing volume overload or pulmonary hypertension—may be similarly predisposed.

Reasons for the lack of reported cases may include:

- Rapid clinical deterioration before diagnosis
- Limited echocardiographic evaluation in large‐animal neonates
- Challenges differentiating functional FO shunting from structural PFO
- Underuse of colour Doppler and contrast echocardiography in young animals

Future veterinary studies should investigate whether FO patency plays a larger role in neonatal outcomes than currently recognized.

Pulmonary hypertension appears to play a central role in the haemodynamic interaction between PDA and PFO by altering right‐sided cardiac pressures and promoting interatrial shunting. Although direct veterinary evidence remains limited, findings from human cardiology literature support the possibility that similar mechanisms may occur in veterinary patients with advanced or untreated PDA.

Further investigation is needed to determine the prevalence, diagnostic implications and clinical significance of concurrent PDA and PFO in veterinary medicine.

## 7 Conclusion

PDA‐induced pulmonary hypertension and altered right‐sided cardiac pressures may theoretically predispose neonatal animals to persistent or reopened PFO, potentially resulting in RA‐to‐LA shunting and cyanosis. Although no clearly documented veterinary case of concurrent PDA and PFO has been identified, the pathophysiological basis is well supported by human literature and warrants further investigation. Increased attention to FO patency during echocardiography of neonates with PDA may enhance diagnostic accuracy and inform treatment strategies.

## Author Contributions

**Amir Mohammad Barati**: conceptualization, literature search, data interpretation, writing – original draft, writing – review and editing, and final approval of the manuscript.

## Funding

The author has nothing to report.

## Conflicts of Interest

The author declares no conflicts of interest.

## Data Availability

Data sharing is not applicable to this article, as no datasets were generated or analysed during the current study.
